# Methane Elimination Using Biofiltration Packed With Fly Ash Ceramsite as Support Material

**DOI:** 10.3389/fbioe.2020.00351

**Published:** 2020-04-22

**Authors:** Meng-Ting Sun, Yu-Zhong Zhao, Zhi-Man Yang, Xiao-Shuang Shi, Lin Wang, Meng Dai, Fei Wang, Rong-Bo Guo

**Affiliations:** ^1^College of Electromechanical Engineering, Shandong Engineering Laboratory for Preparation and Application of High-Performance Carbon-Materials, Qingdao University of Science & Technology, Qingdao, China; ^2^Shandong Industrial Engineering Laboratory of Biogas Production & Utilization, Key Laboratory of Biofuels, Qingdao Institute of Bioenergy and Bioprocess Technology, Chinese Academy of Sciences, Qingdao, China; ^3^Dalian National Laboratory for Clean Energy, Dalian, China

**Keywords:** methane-oxidizing bacteria, immobilization, methane biofiltration, fly ash ceramsite, surface property

## Abstract

Methane is a greenhouse gas and significantly contributes to global warming. Methane biofiltration with immobilized methane-oxidizing bacteria (MOB) is an efficient and eco-friendly approach for methane elimination. To achieve high methane elimination capacity (EC), it is necessary to use an exceptional support material to immobilize MOB. The MOB consortium was inoculated in biofilters to continuusly eliminate 1% (*v/v*) of methane. Results showed that the immobilized MOB cells outperformed than the suspended MOB cells. The biofilter packed with fly ash ceramsite (FAC) held the highest average methane EC of 4.628 g h^–1^ m^–3^, which was 33.4% higher than that of the biofilter with the suspended MOB cells. The qPCR revealed that FAC surface presented the highest *pmoA* gene abundance, which inferred that FAC surface immobilized the most MOB biomass. The XPS and contact angle measurement indicated that the desirable surface elemental composition and stronger surface hydrophilicity of FAC might favor MOB immobilization and accordingly improve methane elimination.

## Introduction

Methane is the second-largest greenhouse gas, which has approximately a 25 times global warming potential than that of carbon dioxide for a 100-year horizon (Veillette et al., 2012; Limbri et al., 2013). Methane is emitted from natural and anthropocentric processes, including wetlands, oceans, forests, paddy fields, manure management, livestock, landfills, coal mines, and biogas upgrading process (Conrad, 2009). 55% of anthropogenic methane emissions hold methane concentrations lower than 3% (*v/v*), and the emitted lean methane is consequently difficult to be treated by the thermal oxidation process (Melse and Van der Werf, 2005; Ramirez et al., 2012a).

**FIGURE 1:**
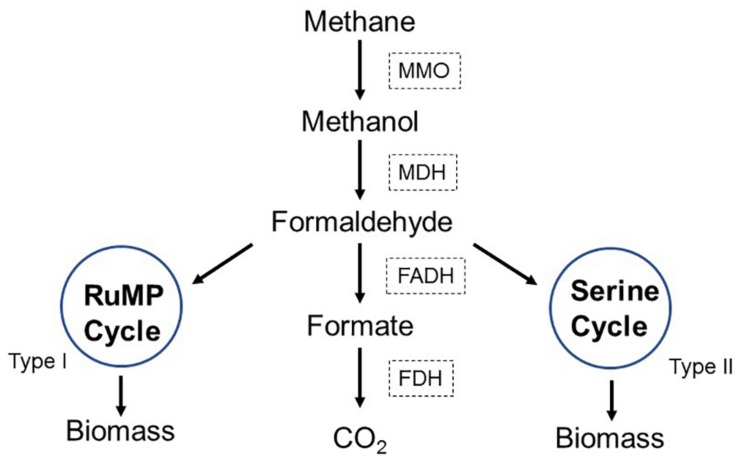
Metabolic pathway of methane in MOB cells.

Methane could be naturally oxidized by methane-oxidizing bacteria (MOB). MOB are widely distributed aerobic microorganisms, which use methane as carbon and energy sources (Im et al., 2011; Ramirez et al., 2012b). As depicted in Figure 1, MOB initially oxidize methane into methanol by methane monooxygenase (MMO), and then methanol is converted into formaldehyde by methanol dehydrogenase (MDH). A part of formaldehyde is oxidized into formate by formaldehyde dehydrogenase (FADH) and subsequently converted into CO_2_ by formate dehydrogenase (FDH). Another part of formaldehyde is assimilated into ribulose monophosphate pathway (RuMP, for Type I methanotrophs) or serine pathway (for Type II methanotrophs) to produce biomass (Kalyuzhnaya et al., 2015). Compared to the thermal oxidation process, the biological conversion is highly qualified for control of methane emission because it permits advantages of mild operation conditions, efficient elimination capacity (EC) and low cost (Ge et al., 2014). MOB consortium is the mixed culture dominated by MOB and simultaneously contains foreign microorganisms. Commonly, MOB are the functional bacteria that oxidize methane, and the accompanying foreign microorganisms supply key nutrients for MOB or remove toxic metabolites. Compared to MOB single strain, MOB consortium usually keeps better growth, higher metabolic activity, and desirable stability, which might accordingly perform more effectively and stably during practical continuous methane elimination (Jiang et al., 2016).

Methane-oxidizing bacteria consortia have been inoculated in biofilters packed with various support materials, such as active carbon, perlite, stones, and polypropylene spheres, to mitigate methane emissions (Kim et al., 2014b; Karthikeyan et al., 2017; Wu et al., 2017). Compared to the suspended MOB cells, the immobilized MOB cells on support materials performed better in methane elimination because of the higher biomass concentration, more excellent metabolic activity and superior tolerance to severe environmental conditions (Cohen, 2000). The bacterial immobilization usually occurs at the interface between cell and support material (Xie et al., 2012). The surface properties of support material, including roughness, porosity, hydrophilicity, charge, and chemical composition can markedly influence on the bacterial immobilization (Farrokhzadeh et al., 2017; La et al., 2018). These surface properties affect MOB immobilization, and further have effects on MOB growth and metabolic activity, and consequently alter the performance of MOB biofiltration. Therefore, the characterization on surface property of support material and the estimation on immobilized MOB biomass are essential, which might be conductive to investigate the effects of surface property of support material on MOB immobilization, and be helpful to figure out the underlying reasons for biofiltration performance in methane elimination.

Ceramsite is a lightweight porous sphere prepared from solid waste, including clay, sludge, shale and fly ash (Merino et al., 2005; Laursen et al., 2006). With advantages of high porosity, inert surface, low cost and excellent durability, ceramsite can provide sufficient area for bacterial adhesion and is an appropriate support material for bacterial immobilization (Qiu et al., 2014; Shi et al., 2015). In our previous study, the MOB consortium has been inoculated in ceramsite to eliminate methane in batch experiments. The results showed that the MOB cells incorporated with black and red ceramsite prepared from fly ash and clay, respectively, kept improved methane ECs, which were 54.4% and 64.4%, respectively, higher than that of the suspended MOB cells during disposing 1% (*v/v*) of methane (Sun et al., 2018c). In this study, to verify the superiority, availability and stability of ceramsite in biofiltration for methane elimination, three lab-scale biofilters packed with fly ash ceramsite (FAC), clay ceramsite (CC) and active carbon, respectively, as support materials were constructed. The methane ECs of biofilters were periodically and continuously tested. The characterization on surface property of support material was carried out, and the immobilized MOB biomass on support material was determined.

## Materials and Methods

### Enrichment Process

The MOB consortium was enriched from the soil, which was harvested from Xiaojianxi Landfill in Qingdao, Shandong, China. The enrichment processes were in compliance with the reported methods, and the nitrate mineral salts (NMS) medium with pH of 6.8 was used to provide nutrient elements for MOB (Sun et al., 2018c). Before inoculation, the MOB consortium was cultivated under methane concentration of 20% (*v/v*) at 25°C with continuous shaking of 140 rpm. The MOB consortium was successionally subcultured every 3 days.

### Preparation and Characterization of Support Material

The FAC and CC were purchased from the Octagon Water Purification and Building Material Factory (Henan, China) as support materials. The active carbon (AC, AKE, Foshan, Guangdong, China) was also regarded as a kind of support material. The FAC, CC, and AC were milled into particles and sieved to the size of 2.0–3.0 mm. To remove the biomass and ashes, the particles were boiled for 1 h in distilled water and washed five times by distilled water, and subsequently dried at 105°C for 48 h.

The X-ray photoelectron spectroscopy (XPS) for support material was carried out by a multifunctional imaging electron spectrometer (ESCALAB250Xi, Thermo Fisher Scientific, Waltham, Massachusetts, United States). To evaluate surface hydrophilicity of support material, the water contact angle on surface of support material was measured by a contact angle meter (XG-CAM, XYCXIE, Shanghai, China).

### Biofilter Configuration and Test

The biofilters were fabricated by Haiyanyakeli Co., Ltd. (Qingdao, Shandong, China). The biofilter configuration is illustrated in Supplementary Figure S1. Four hollow biofilters with heights of 40 cm and diameters of 4 cm were made from polymeric methyl methacrylate (PMMA). One biofilter without support material was named MC. Three biofilters named MFAC, MCC, and MAC were filled with FAC, CC, and AC, respectively, to the heights of 25 cm. Each biofilter was inoculated with 150 mL of MOB consortium slurry (2.47 ± 0.06 g L^–1^) and then filled with NMS medium to the height of 35 cm. To fully mix up the content, each filled biofilter was gently shaken by hand for 5 min. Butyl rubber stoppers were used to seal the feeding and drainage ports to make biofilters gas tight. To provide adequate nutrient elements, 0.1 L of liquid was daily drained from each biofilter through drainage port and an identical volume of fresh NMS medium was added via feeding port (Ramirez et al., 2012b). The biofilters were incubated at 25°C. The gas mixture of methane, carbon dioxide and air at a mixing ratio of 1:15:84 (*v/v/v*) was prepared by Heli Gas Co., Ltd. (Qingdao, Shandong, China) and was fed through gas inlet on the bottom of each biofilter at a gas flow rate of 0.9 L h^–1^. The configuration and operating parameters of biofilters are listed in Table 1.

**TABLE 1:** The configuration and operating parameters of biofilters.

**Parameter**	**Value**
Empty biofilter volume (m^3^)	0.0005
Biofilter bed volume (m^3^)	0.0003
Gas flow rate (L h^–1^)	0.9
Gas mixing ratio (CH_4_/CO_2_/Air)	1:15:84
Temperature (°C)	25
Empty bed residence time (EBRT, min)	20

The gas samples were periodically gathered from each gas outlet and the methane concentrations of samples were determined by gas chromatography (GC-2014, Shimadzu, Nakagyo-ku, Kyoto, Japan) equipped with a flame ionization detector and a WAX-DA column (30.0 m × 0.32 mm × 0.50 μm). Nitrogen was used as carrier gas, and temperatures for detector, vaporizer and oven were 150, 150, and 120°C, respectively. The methane EC was defined as the mass of methane eliminated by MOB immobilized on per weight of support material during per hour, and was calculated using the Equation 1:

$${\text{EC(g h}}^{-1}{\text{m}}^{-3})=\text{R}\times \text{M}{}_{\text{c}}\times ({\text{C}}_{\text{in}}-{\text{C}}_{\text{out}})/({\text{V}}_{\text{b}}\times {\text{V}}_{\text{m}})$$

where R was the inlet gas flow rate (L h^–1^), M_c_ was the molar mass of CH_4_ which was 16 g mol^–1^, C_in_ and C_out_ were the inlet and outlet methane concentrations (%), respectively, V_b_ was the biofilter bed volume (m^3^) and V_m_ was the gas molar volume which was 22.4 L mol^–1^.

### Scanning Electron Microscopy (SEM)

Ten pieces of support materials were collected from MFAC, MCC, and MAC at the 20th day of biofilter operation. The particles were rinsed with distilled water for three times to remove the unattached bacterial cells from their surfaces. The samples were immersed in 4% of glutaraldehyde for 5 h at 4°C, and washed three times by 0.2 mol L^–1^ of phosphate buffer (pH 7.0) for 15 min. Subsequently, the samples were dehydrated by 50%, 70%, 80%, 90%, and 95% of ethanol by submerging samples at each concentration for 15 min, and then dehydrated two times by 100% of ethanol for 20 min. The treated samples were freeze-dried by a lyophilizer (10 N, SCIENTZ, Ningbo, Zhejiang, China) for 24 h, and sputter-coated with a thin layer of metallic gold (E-1010, Hitachi, Chiyoda, Tokyo, Japan). Microphotographs of the samples were carried out by a scanning electron microscope (SU8010, Hitachi, Chiyoda, Tokyo, Japan).

### Quantitative PCR (qPCR) Analysis

The biomass of MOB immobilized on support material was tested through the qPCR analysis based on *pmoA* gene detection. The *pmoA* gene encodes a subunit of particulate methane monooxygenase enzyme (pMMO) in MOB cells, and the copy number of *pmoA* gene could convincingly reflect the MOB biomass (Kolb et al., 2003; He et al., 2012). At the 20th day of biofilter operation, triplicate samples, each contains 0.25 g of support material, were randomly harvested from each biofilter and stored at −80°C in a freezer before DNA extraction. The DNA samples were extracted by FastDNA Spin kit for Soil (TIANGEN, Beijing, China). 0.8% (*w/v*) agarose gel was used to check the quality of DNA samples. Afterward, Qubit 2.0 Fluorometer (Invitrogen, Carlsbad, California, United States) was applied to quantify DNA samples. The *pmoA* gene in each DNA sample was amplified by forward primer A189f (5′-GGNGACTGGGACTTCTGG-3′) and reverse primer Mb661r (5′-CCGGMGCAACGTCYTTACC-3′) (Martineau et al., 2010; Sun et al., 2018a).

The qPCR assays were conducted by a Rotor-Gene Q real-time PCR cycler (ABI7500, Applied Biosystems, Waltham, United States). Each qPCR reaction with 18 μL of total volume contained 10 μL of 2 × MasterMix (Cw0716, CWBIO, Beijing, China), 2 μL of diluted template DNA, 0.5 μL of A189f forward primer (10 μmol L^–1^), 0.5 μL of Mb661r reverse primer (10 μmol L^–1^), and 5 μL of sterile distilled water. The protocol for qPCR was as follow: initial denaturation at 90°C for 30 s, followed by 40 cycles of 95°C for 5 s, 60°C for 40 s; the Fluorescence signal was obtained after each cycle at 60°C for 1 min; and the melt curve obtained from 60°C to 99°C with a rate of 0.05°C s^–1^. The qPCR assays of successive 10-fold dilutions (10^1^∼10^5^) of plasmid Puc-T inserted with *pmoA* gene were performed in triplicate to obtain the standard curve (Sun et al., 2018a).

## Results

### Surface Property of Support Material

The XPS and contact angle measurement were conducted to detect the surface elemental composition and the surface hydrophilicity of support material, respectively. The surface elemental compositions are shown in Table 2. The ceramsite surface was dominated by O, which took 31.66% and 29.02% on surfaces of FAC and CC, respectively. Si was another predominant element of ceramsite surface, which occupied 24.45% and 26.22% on surfaces of FAC and CC, respectively. The C was the most principal element on AC surface and with a percentage of 87.97%, while O merely accounted for 7.73% on AC surface. Fe content occupied with 0.89%, 1.62%, and 0.21% on surfaces of FAC, CC and AC, respectively. For Al content, it was identified with 14.29%, 15.58% and 0.81% on surfaces of FAC, CC, and AC, respectively.

**TABLE 2:** The surface properties of support materials.

	**Surface elemental composition (atom, %)**							
**Support material**								**Contact angle (°)**
	**C**	**N**	**O**	**S**	**Si**	**Al**	**Fe**	
FAC	25.49	1.69	31.66	1.53	24.45	14.29	0.89	42.0 ± 4.4**°**
CC	25.58	1.43	29.02	0.53	26.22	15.58	1.62	46.7 ± 1.5**°**
AC	87.97	1.91	7.73	0.29	1.07	0.81	0.21	131.7 ± 0.6**°**

The water contact angle on support material could be calculated using the reported method and reflects the hydrophilicity of material surface (Wang et al., 2015). As shown in Table 2 and Supplementary Figure S2, the water contact angles on surfaces of FAC, CC, and AC were 42.0 ± 4.4°, 46.7 ± 1.5°, and 131.7 ± 0.6°, respectively. With a smaller water contact angle, the material might hold a stronger hydrophilic surface (Sun et al., 2018a). Evidently, compare to AC surface, the ceramsite surface presented superior hydrophilicity, and the FAC surface possessed the strongest hydrophilicity among three support materials.

### Methane Elimination Performance

**FIGURE 2:**
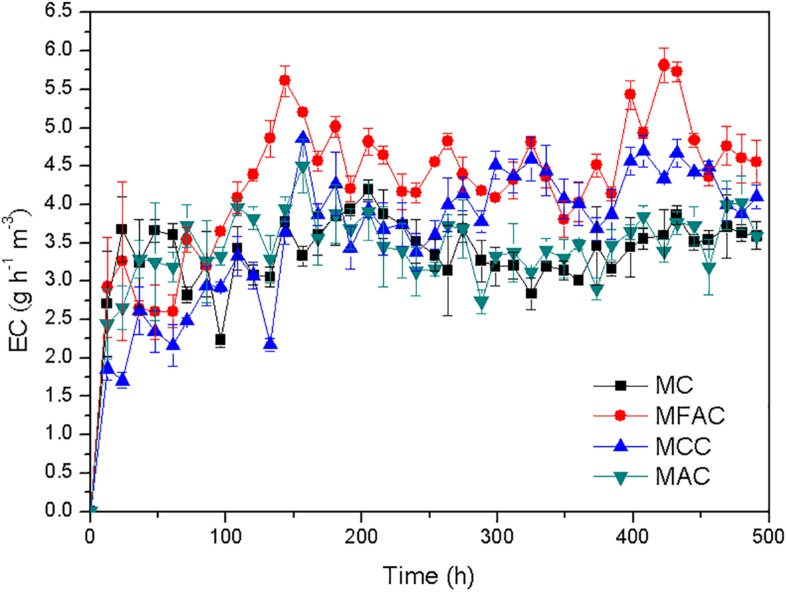
Methane ECs of biofilters packed with different support materials.

As illustrated in Figure 2, the methane EC of MFAC dramatically increased during the initial period and achieved a value of 5.610 g h^–1^ m^–3^ at 143.5 h, and subsequently maintained at a stable level, which indicated that the biofilter entered into a stable operation. The methane EC of MCC increased with fluctuations during the initial period and reached a value of 4.860 g h^–1^ m^–3^ at 156.5 h, and afterward went into a stable level. The methane ECs of MC and MAC robustly increased during a short initial period, and reached 3.234 g h^–1^ m^–3^ and 3.287 g h^–1^ m^–3^ at 36.5 h, respectively, and then turned to be stable. Among four biofilters during the whole operation duration, the highest methane EC was achieved by MFAC at 422.5 h, which was 5.814 g h^–1^ m^–3^. For MC, MCC and MAC, the highest methane ECs during their individual operations were 4.194, 4.860 and 4.505 g h^–1^ m^–3^, respectively. For each biofilter, the initial period with ever-increasing methane EC could be regarded as the start-up phase, which was mainly for the bacterial adhesion and the biofilm formation on support material. After that, four biofilters successively went into stable operations and stably eliminated methane. The average methane EC after 100 h was estimated to reflect the methane elimination ability of each biofilter. The average methane ECs of MFAC, MCC, and MAC attained 4.628, 3.989 and 3.556 g h^–1^ m^–3^, which were 33.4%, 15.0%, and 2.5% higher than that of MC (3.469 g h^–1^ m^–3^), respectively. The results demonstrated that MFAC was the most effective methane eliminator, and followed by MCC, MAC, and MC.

### SEM of MOB Immobilized on Support Material

**FIGURE 3:**
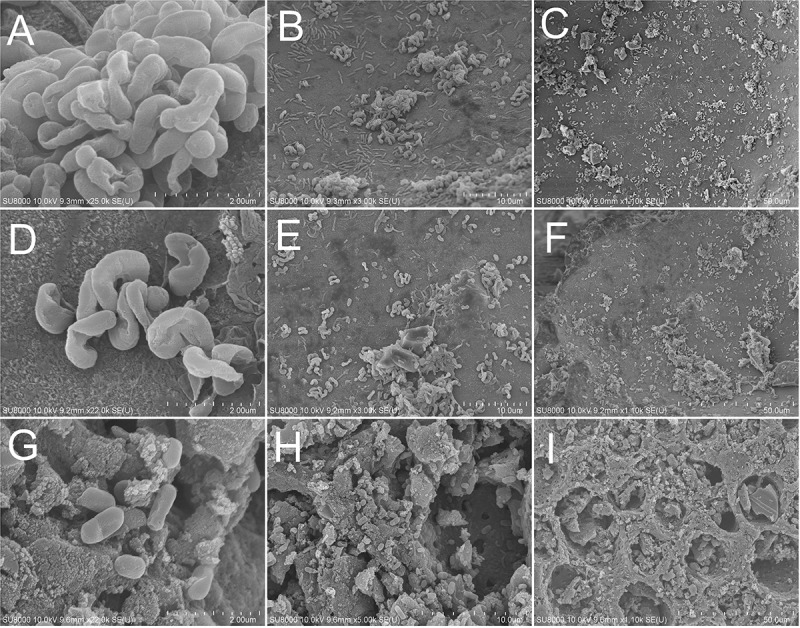
SEM images of MOB immobilized on support materials. **(A,B,C)** for FAC; **(D,E,F)** for CC; **(G,H,I)** for AC.

The SEM images of support materials harvested from MFAC, MCC, and MAC at the 20th day showed that, a great number of non-motile, short and slightly curved rod bacterial cells with the width of 0.5–1.0 μm and the length of 1.0–2.0 μm adhered to surfaces of FAC, CC, and MAC, which were morphologically similar to the reported MOB cells (Figure 3; Dedysh et al., 2002). It implied that MOB immobilization has been achieved on surfaces of support materials in biofilters.

### Evaluation on Biomass of MOB Immobilized on Support Material

**FIGURE 4:**
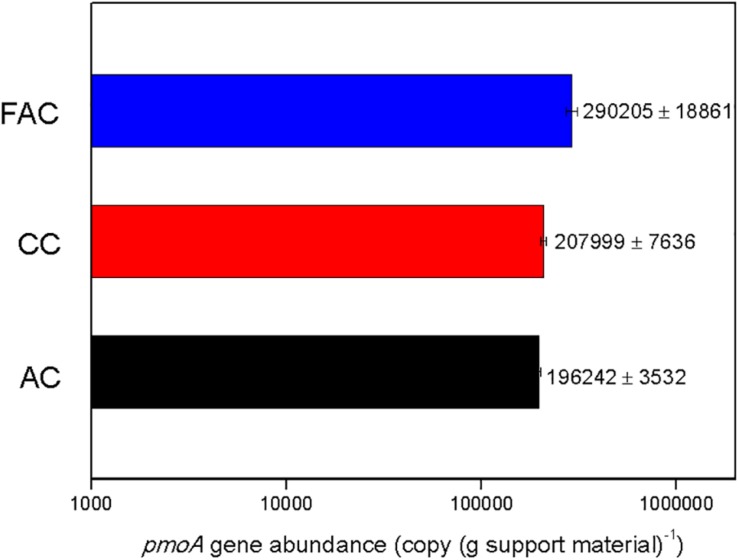
*pmoA* gene abundance on surface of support material.

To investigate the biomass of MOB immobilized on surface of support material, the qPCR analysis based on *pmoA* gene detection was conducted. The *pmoA* gene abundance on surface of support material is illustrated in Figure 4. The *pmoA* gene copy numbers on surfaces of FAC, CC, and AC were 290205 ± 18861, 207999 ± 7636, and 196242 ± 3532 copies (g support material)^–1^, respectively. The FAC surface held the highest *pmoA* gene abundance, which were 39.5% and 47.9% higher than that on CC surface and AC surface, respectively. The results inferred that FAC surface immobilized the most MOB biomass.

## Discussion

### Surface Property of Support Material

As shown in Table 2, FAC surface possessed the highest O content, followed by CC surface. Compared to ceramsite, AC surface had the poorest O content. The results indicated that FAC surface might hold the greatest number of O-containing groups among three support materials. The sufficient O-containing groups on surface could possibly lead to a strong surface hydrophilicity (Bao and Dai, 2014). Both FAC surface and CC surface mainly contained O and Si, which indicated that SiO_2_ might be the main framework of ceramsite. The CC surface kept the highest Fe content, which might be the reason for the red color of its appearance, and also indicated that CC surface possibly contained a great amount of Fe_2_O_3_. Evidently, ceramsite surface contained higher contents of metal elements than AC surface, including Fe and Al, which inferred that ceramsite surface presumably consisted of a variety of metal oxides. In aqueous environment, the metal hydroxides might be formed on ceramsite surface instead of metal oxides, and the covalent bonds could consequently be formed between bacterial cells and metal hydroxides, which might facilitate the bacterial adhesion and immobilization (Cohen, 2000). Additionally, the sufficient metal elements might possibly provide additional mineral elements for bacteria and consequently be beneficial for their growth and metabolic activity. Collectively, according to the results of surface elemental compositions of support materials, ceramsite surface held higher contents of O and metal elements compared to AC surface, which might lead to the stronger hydrophilicity and sufficient metal oxides on ceramsite surface, and accordingly exhibited preferable biocompatibility for bacterial immobilization.

The FAC surface with the smallest water contact angle kept the strongest hydrophilicity, followed by CC surface, and AC surface comparatively showed the largest water contact angle and was revealed to be highly hydrophobic (Table 2 and Supplementary Figure S2). The hydrophilic surface could favor the adhesion of bacteria with hydrophilic cell surfaces, while the hydrophobic surface could favor the adhesion of bacteria with hydrophobic cell surfaces (Muller et al., 2008). For bacteria with hydrophilic cell surfaces, the FAC surface with the strongest hydrophilicity might favor the bacterial adhesion, and thereby permitted the favorable biocompatibility for bacterial immobilization.

### Methane Elimination and MOB Immobilization Performances

The average methane ECs of MFAC, MCC, and MAC were higher than that of MC, which suggested that the biofilters packed with support materials outperformed in methane elimination than biofilter with the suspended MOB cells (Figure 2). The SEM images of support materials collected from biofilters revealed that the MOB immobilization occurred on surfaces of FAC, CC, and AC (Figure 3). Commonly, compared with the suspended bacteria, the immobilized bacteria exhibit higher biomass density, favorable metabolic activity, and preferable resistance to severe environmental conditions (Cohen, 2000). Accordingly, the better performances of MFAC, MCC, and MAC in methane elimination could be attributed to the MOB immobilization occurred on surfaces of FAC, CC, and AC, respectively. Apart from the improving effect of MOB immobilization, the high porosity and ample pore volume of support materials might be another improving factor. The gas could enter into these pores, which might prolong the gas retention time in biofilters and enhance the contact chance between MOB cells and methane, and consequently further promoted methane elimination (Karthikeyan et al., 2017; Wu et al., 2017). Similarly, in existing studies, the active carbon prepared from biogas digestate has been used as support material to immobilize MOB consortium in a biofilter to dispose 0.9% (*v/v*) of methane, and reached a methane EC of 2.08 g h^–1^ m^–3^, which was almost four folds of that of the suspended MOB cells (Wu et al., 2017); A lad-scale methane biofilter regarding tobermolite as support material was installed to mitigate 5% (*v/v*) of methane, and the methane ECs ranged from 27.72 g h^–1^ m^–3^ to 28.96 g h^–1^ m^–3^ were obtained (Kim et al., 2014a) (Table 3).

**TABLE 3:** Study cases of MOB biofilters.

**Support material**	**CH_4_ concentration (%, *v/v*)**	**Empty biofilter volume (m^3^)**	**Biofilter bed volume (m^3^)**	**Gas flow rate (Lh^–1^)**	**Empty bed residence time (EBRT, min)**	**Methane EC (g h^–1^ m^–3^)**	**References**
Volcanic	0.9	0.0014	0.0007	0.0006	70	2.56	Sun et al., 2018b
Active carbon	0.9	0.0014	0.0007	0.0006	70	2.08	Wu et al., 2017
Sponge	0.9	0.0014	0.0007	0.0006	70	3.52	Sun et al., 2018b
Perlite	5	0.005	0.005	0.015	20	46.40	Kim et al., 2014b
Tremolite	5	0.005	0.005	0.015	20	27.72∼28.96	Kim et al., 2014a
Glass	1	0.0015	0.001	0.0009	67	2.88	Karthikeyan et al., 2017
Polypropylene	1	0.0015	0.001	0.0009	67	3.32	Karthikeyan et al., 2017
Coal	1	0.0044	0.0039	0.096	2.4	19.20	Limbri et al., 2013
autoclaved aerated concrete	0.1	0.0051	0.0026	0.2	0.8	5.28	Ganendra et al., 2015
Suspended MOB	1	0.0005	0.0003	0.9	20	3.469	This work
FAC	1	0.0005	0.0003	0.9	20	4.628	This work
CC	1	0.0005	0.0003	0.9	20	3.989	This work
AC	1	0.0005	0.0003	0.9	20	3.556	This work

The methane ECs of MFAC and MCC were higher than that of MC, which revealed that the biofilters packed with ceramsite behaved more efficiently than that with AC during methane elimination. Among these biofilters, the MFAC was the most effective methane eliminator (Figure 2). The qPCR analysis convincingly reflected the biomass of MOB immobilized on the surfaces of FAC, CC, and AC, and revealed that FAC surface possessed the highest *pmoA* gene abundance and was markedly higher than that on CC surface and AC surface, which demonstrated that FAC surface possibly immobilized the most MOB biomass among three support materials (Figure 4). The most MOB biomass on FAC surface might be possibly ascribed to the preferable surface property of FAC, which favored MOB adhesion and immobilization. With the most immobilized MOB biomass on FAC surface, the MFAC accordingly achieved the best performance in methane elimination.

Taking the results of XPS and contact angle measurement into consideration, the surface elemental composition and hydrophilicity of support material might be the influential factors on MOB immobilization. According to the surface elemental compositions of support materials, the ceramsite surface contained higher contents of O and metal elements than AC surface, which implied that FAC and CC kept abundant metal oxides on their surfaces (Table 2). The sufficient metal oxides on ceramsite surface could be converted into metal hydroxides in aqueous environment, and consequently the covalent bonds could be formed between metal hydroxides and MOB cells, which were beneficial to MOB immobilization and resulted in more MOB biomass on ceramsite surface (Cohen, 2000). Additionally, Fe has been reported to be an essential trace element for MOB growth and metabolism (Hirayama et al., 2011; Han et al., 2013). With higher Fe content, the ceramsite might possibly provide additional Fe element for MOB growth and metabolic activity, and could consequently contribute to the promotion of methane elimination. Accordingly, compared to AC, the favorable surface elemental composition of ceramsite might be an underlying reason for better performances of MFAC and MCC in MOB immobilization and methane elimination. Li and Logan have prepared 11 types of metal oxide-coated (Si-m, Si-a, Si-Sn, TiO_2_, SnO_2_, A1_2_O_3_, Fe_2_O_3_, Co/Fe/Cr/O, SnO_2_:Sb, SnO_2_:F, and Ti/Fe/O) glasses to immobilize eight bacteria, and the results indicated that the metal oxides-coated glass surfaces could facilitate bacterial adhesion and persisted more cell adhesion number than the uncoated glass surface (Li and Logan, 2004).

The surface hydrophilicity could also significantly affect the efficiency of bacterial immobilization. It was mentioned that the bacteria with hydrophilic cell surfaces were likely to adhere to the hydrophilic material surface (Muller et al., 2008). The microbial community analysis performed in the previous study unveiled that the functional methanotrophs in inoculated MOB consortium were genera *Methylomonas* and *Methylocaldum* (Supplementary Figure S3). These two genera use methane as sole carbon and energy sources for growth, which might be the major methane oxidizers in MOB consortium. They were assigned to Type I methanotrophs (Hirayama et al., 2011; Wei et al., 2016). Starostina et al. (1997) argued that the cell surfaces of Type I methanotrophs were mainly hydrophilic. In this study, the contact angle measurement revealed that FAC presented the strongest hydrophilic surface among three support materials (Table 2 and Supplementary Figure S2). With the strongest surface hydrophilicity, FAC might favor the adhesion of hydrophilic methanotrophs genera *Methylomonas* and *Methylocaldum*, and consequently led to more MOB cells immobilized on FAC surface. The qPCR analysis also indicated that FAC surface kept the greatest copy number of *pmoA* gene, which implied that FAC surface might immobilize the most MOB biomass among three support materials (Figure 4). Due to the most MOB biomass on FAC surface, the MFAC consequently achieved superior methane EC than MCC and MAC. Accordingly, compared to CC and AC, the stronger surface hydrophilicity of FAC might be another underlying reason for the better performance of MFAC in methane elimination. Bao and Dai (2014) suggested that the improved surface hydrophilicity of carbon fiber could favor microbial immobilization on its surface, and they used HNO_3_ to modify carbon fiber and promote its surface hydrophilicity, and the modified carbon fiber kept a microbial immobilization ratio which was 93.9% higher than that of the unmodified carbon fiber.

In this work, a MOB consortium was inoculated in biofilters packed with FAC, CC, and AC, respectively, to continuously eliminate 1% (*v/v*) of methane. The biofilters with immobilized MOB cells exhibited higher methane ECs than that of the biofilter with the suspended MOB cells. The MFAC and MCC, the biofilters with MOB inoculated in ceramsite FAC and CC, both behaved better than that in AC due to their more favorable surface elemental compositions, which benefited for MOB immobilization. The MFAC, the biofilter with MOB immobilized on FAC, kept the highest average methane EC of 4.628 g h^–1^ m^–3^ among four biofilters, which was 33.4% higher than that of the biofilter with the suspended MOB cells. The qPCR analysis inferred that FAC surface immobilized the most MOB biomass among three support materials, which might be owed to the strong surface hydrophilicity of FAC that possibly in favor of MOB immobilization. Collectively, the preferable surface elemental composition and strong surface hydrophilicity of FAC possibly favored MOB immobilization and consequently improved methane elimination of biofilter. The results in this study could provide a few suggestions for future work on methane biofiltration, which are listed as follows:

(1) To improve methane EC, increasing immobilized MOB biomass on support material might be feasible;
(2) The selection of support material is critical, and the support  material  with  favorable  surface  biocompatibility might be an appropriate choice for supporting MOB immobilization;
(3) In methane biofiltration, the support materials with strong surface hydrophilicity and hydrophobicity, might be qualified for MOB cells with hydrophilic and hydrophobic surfaces, respectively;
(4) To facilitate MOB immobilization, the support material keeping abundant metal oxides on surface could be a desirable candidate;
(5) The adequate O-containing groups on support material surface could favor MOB immobilization;
(6) Support material with exposed porous structure on surface is strongly recommended, which might benefit for MOB immobilization as well prolong methane retention time in biofilter.

Due to the remarkable advantages, the methane biofiltration hold a huge application potential in eliminating methane and mitigating global warming. This work intended to optimize EC of methane biofiltration via applying exceptional support material with desirable surface property. The FAC, CC, and AC were utilized as support materials for MOB immobilization to construct methane biofilters, the methane elimination was significantly improved by FAC and moderately promoted by CC and AC. To figure out the underlying reasons for diverse performances of different support materials, the immobilized MOB biomass on support material was investigated, meanwhile the surface elemental composition and hydrophilicity of support material were characterized, thus the correlation among surface property, immobilized MOB biomass and methane EC of biofilter was elaborately discussed. This work firstly discussed the influential mechanism of surface property of support material on MOB immobilization and methane elimination in lab-scale continuous experiments, which might provide reliable evidences and helpful advices for support material selection for methane biofiltration.

Certainly, this study also has few limitations. On the one hand, the first, second, and third emission standards of methane in China were 0.5% (*v/v*), 1.0 (*v/v*), and 1.0% (*v/v*), respectively, however, the lowest average outlet methane concentration in this work reached to 0.78% (*v/v*) by MFAC, which failed to meet the first emission standard, and consequently the further improvement work on methane biofilter needs to be undertaken. According to the results in this work, the optimization on surface biocompatibility of support material via chemical modification might possibly be a feasible approach to facilitate MOB immobilization and methane elimination. On the other hand, the superiority and stability of ceramsite have been tested in batch and lab-scale continuous experiments in our completed studies, which need to be further verified in methane biofiltration with a larger scale in practical applications, thereby the scale-up on methane biofiltration using ceramsite as support material and its practical applications in actual methane emission sites need to be proceeded in further study.

## Conclusion

The exceptional support material with biocompatible surface property could efficiently immobilize MOB cells and make MOB achieve high biomass density, metabolic activity, desirable stability and resistance, which is helpful to reach a decent methane EC during biofiltration. The FAC, a kind of ceramsite prepared from fly ash, could effectively immobilize MOB cells due to its favorable surface elemental composition and strong surface hydrophilicity, and might be an exceptional support material for methane biofiltration. The use of FAC support material in methane biofiltration might facilitate the industrial and scaled application of MOB in methane elimination.

## Data Availability Statement

The raw data supporting the conclusion of this article will be made available by the authors, without undue reservation, to any qualified researcher.

## Author Contributions

M-TS performed the experiments and wrote the manuscript. Y-ZZ designed the configuration and operation of biofilters. Z-MY, FW, and R-BG gave the ideas for this project and help in the case of scientific problems. X-SS, MD, LW, FW, and R-BG supervised and revised the manuscript.

## Conflict of Interest

The authors declare that the research was conducted in the absence of any commercial or financial relationships that could be construed as a potential conflict of interest.
