# Supplementary material for: Methane Elimination Using Biofiltration Packed With Fly Ash Ceramsite as Support Material
Source: Front Bioeng Biotechnol. 2020 Apr 22;8:351. doi: 10.3389/fbioe.2020.00351 (PMC7188830; doi:10.3389/fbioe.2020.00351)
Supplement: Supplementary file 1 [file Data_Sheet_1.pdf]

Supplementary Information

Methane elimination using biofiltration packed with fly ash ceramsite as support material

Meng-Ting Sun [1], Yu-Zhong Zhao [2], Zhi-Man Yang [2], Xiao-Shuang Shi [2], Lin Wang [2], Meng Dai [2], Fei Wang [1], Rong-Bo Guo [2,3*]*

1 Shandong Engineering Laboratory for Preparation and Application of High-performance Carbon-materials, College of Electromechanical Engineering, Qingdao University of Science & Technology, Qingdao, China, 2 Shandong Industrial Engineering Laboratory of Biogas Production & Utilization, Key Laboratory of Biofuels of Chinese Academy of Sciences, Qingdao Institute of Bioenergy and Bioprocess Technology, Chinese Academy of Sciences, Qingdao, China, 3 Dalian National Laboratory for Clean Energy, Dalian, China

Correspondence:
Rong-Bo Guo
guorb@qibebt.ac.cn
Fei Wang
elliot_wang@qust.edu.cn

First author:
Meng-Ting Sun
sunmt@qust.edu.cn

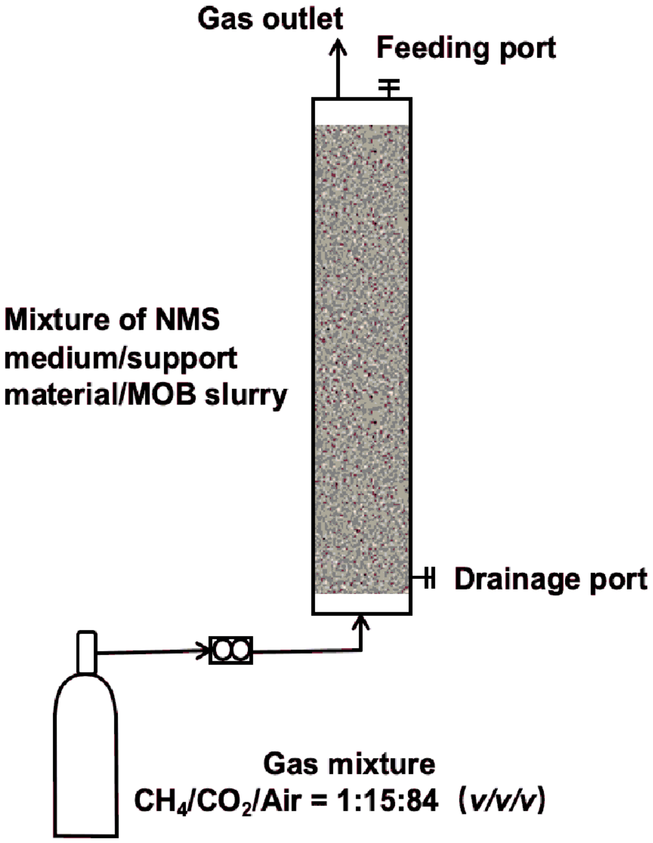

FIGURE S1 | Schematic representation for biofilter configuration.

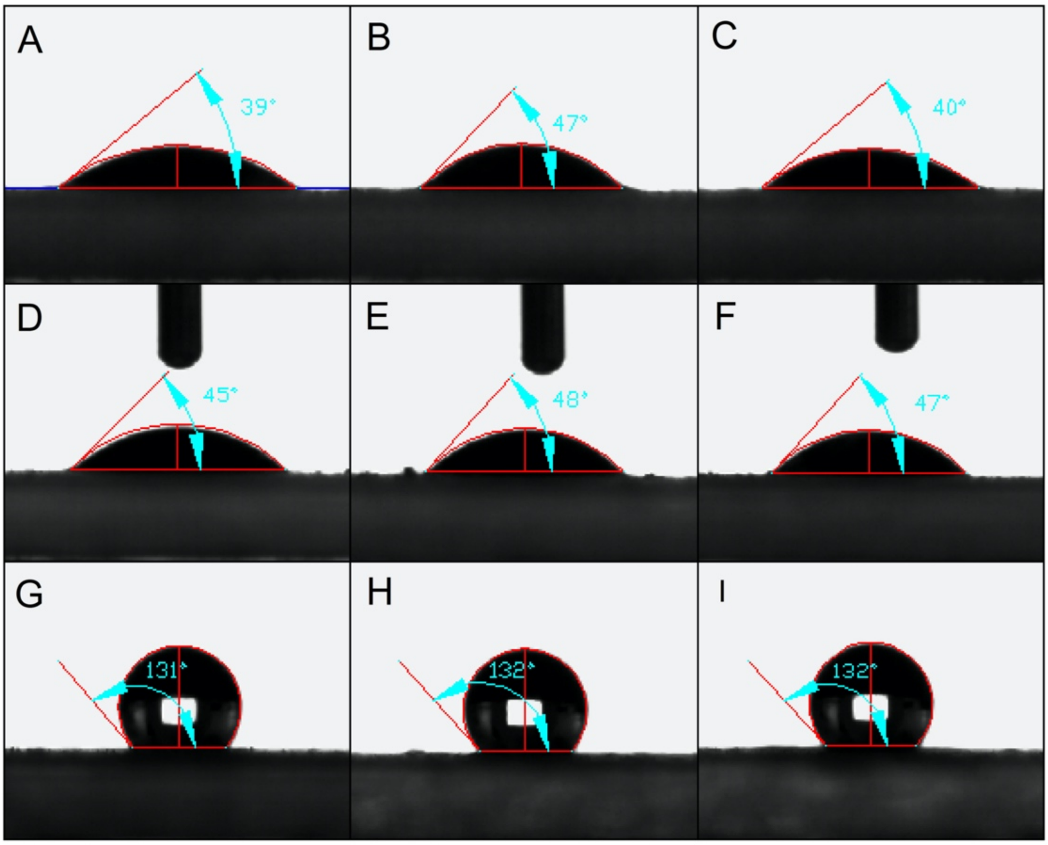

FIGURE S2 | Contact angles of distilled water on support materials. **(A), (B), (C)** for FAC; **(D), (E), (F)** for CC; **(G), (H), (I)** for AC.

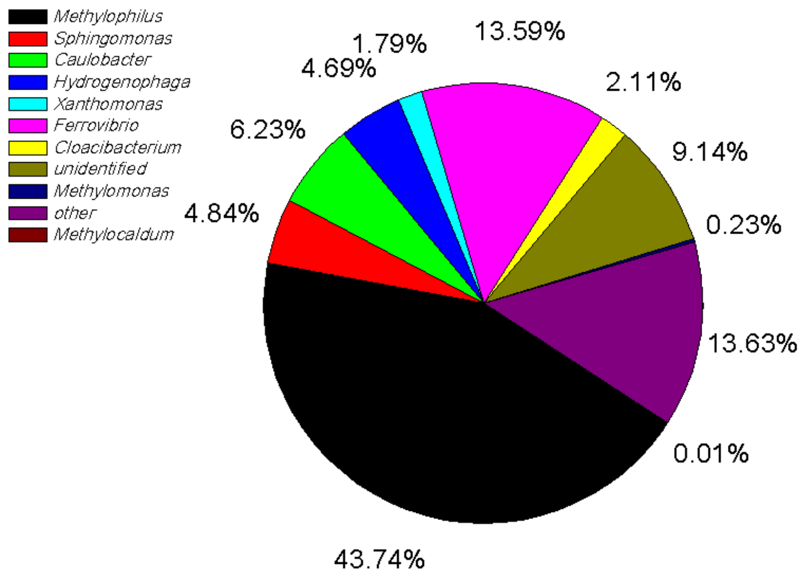

FIGURE S3 | Microbial community composition of MOB consortium at genus level; reproduced from Sun et al., (2018a) with the permission from Wiley-VCH.
